# The Role of GI Peptides in Functional Dyspepsia and Gastroparesis: A Systematic Review

**DOI:** 10.3389/fpsyt.2020.00172

**Published:** 2020-03-18

**Authors:** Karen Van den Houte, Emidio Scarpellini, Wout Verbeure, Hideki Mori, Jolien Schol, Imke Masuy, Florencia Carbone, Jan Tack

**Affiliations:** Translational Research Center for Gastrointestinal Diseases, University of Leuven, Leuven, Belgium

**Keywords:** functional dyspepsia, gastroparesis, gastrointestinal peptides, cholecystokinin, glucagonlike peptide 1, peptide YY, motilin, ghrelin

## Abstract

Functional dyspepsia (FD) and gastroparesis (GP) are common disorders of the upper gastrointestinal tract. The pathophysiology of these conditions is likely to be heterogenous, and factors such as altered motility, sensitivity and response to nutrition have been identified as putative underlying mechanisms. Motility, sensitivity as well as responses to nutrition can be influenced or mediated by peptide hormones and serotonin released from the gastrointestinal mucosa. This review summarizes the role of GI peptides in functional dyspepsia and gastroparesis. In most studies, the levels of somatostatin, ghrelin, and motilin did not differ between healthy volunteers and FD or GP patients, but higher symptom burden was often correlated with higher peptide levels. Ghrelin and motilin receptor agonists showed promising results in improvement of the gastric emptying, but the link with improvement of symptoms is less predictable. Serotonin agonists have a potential to improve symptoms in both FD and idiopathic gastroparesis. Drugs acting on the GLP-1 and on the PYY receptors deserve further investigation. There is a need for systematic large scale studies.

## Introduction

### Functional Dyspepsia

Functional dyspepsia (FD), defined as “epigastric symptoms affecting daily life, such as postprandial fullness, early satiation, epigastric pain and burning, in the absence of underlying organic abnormalities” (1), is an extremely common functional gastrointestinal disorder. In the general population, the prevalence of FD is found to be up to 21% (2, 3). Although only a minority of *H. pylori* infected patients remain asymptomatic after successful eradication therapy, patients reporting *helicobacter pylori*-associated dyspeptic symptoms are now being recognized as a separate entity referred to as *H. pylori* associated dyspepsia (1, 4, 5).

To facilitate the management of FD, the Rome Consensus subdivided FD into two subtypes: Postprandial Distress Syndrome (PDS) (60%) characterized by meal-related symptoms, such as postprandial fullness, early satiation, postprandial epigastric pain and other symptoms triggered by food ingestion, and Epigastric Pain Syndrome (EPS) (20%) characterized by epigastric pain and burning (4, 6). Approximately 20% of FD patients overlaps between PDS and EPS.

FD is extremely common, with estimates of 10–30% prevalence in the general population, and is associated with substantial medical care costs and a considerable health economic impact (7–9). A proportion of 20–25% of the patients with severe and refractory GI symptoms also have psychosocial co-morbidities such as anxiety, depression or somatization and severely impaired daily functioning (about 10% of these patients have work disability). Somatization, namely multiple stress-related symptoms of unknown origin resulted to be the most important risk factor for impaired QOL in patients with severe functional dyspepsia (10). This FD subgroup is often referred to advanced care, which may be associated with even higher health economic costs (11).

Finally, FD patients also show an important degree of overlap with gastro-esophageal reflux disease (GERD) (12, 13) and irritable bowel syndrome (IBS), and are, thus, often misclassified.

### Gastroparesis

Gastroparesis is characterized by delayed gastric emptying and by upper gastrointestinal symptoms (nausea, vomiting, abdominal pain, early satiety, bloating) in the absence of mechanical obstruction (14). Two of the most common types of gastropareses are idiopathic gastroparesis and diabetic gastroparesis (15). Gastroparesis can also be a complication of upper gastrointestinal surgery, neurological disease, collagen vascular disorders, viral infections, or drugs use (16). It is associated with a major impact on the patients' quality of life and substantial social and health economic costs (17).

### Gastrointestinal Peptides

In the classical pathophysiological model, functional gastrointestinal disorders (FGIDs) are considered heterogeneous conditions, and symptoms are attributed to a combination of motility disturbances, visceral hypersensitivity, low grade mucosal immune activation, and altered processing of gut-brain signals (18). This is based on the presence of impaired gastric storage and emptying function in FD and gastroparesis, as well as findings of visceral hypersensitivity and increased levels of depression, somatization and anxiety, which are considered markers of altered gut-brain interaction (19–21).

Recent research has focused on visceral hypersensitivity as a common mechanism determining symptom severity and impact across several functional gastrointestinal disorders (19). To date, the focus of research has mainly been on hypersensitivity to mechanical stimuli, studied by balloon distention (22). However, there is increasing evidence for a role for visceral hypersensitivity to specific nutrients as well, suggested amongst other by the observation that FODMAPs induce symptoms and the observation that specific nutrients induce local immune activation in irritable bowel syndrome (IBS) patients but not in health (23, 24).

The gastrointestinal mucosa expresses a wide range of chemosensing receptors, which detect the presence and nature of nutrients in the lumen (25, 26). Nutrients are mainly sensed in the duodenum and jejunum, and initiate an avalanche-effect by releasing gut peptides from entero-endocrine cells into the blood stream. The brain receives these signals through activation of the vagus nerve or directly via the fenestrated blood brain region, the area postrema (25, 26).

There is recent evidence of nutrient-specific enhanced release of gut peptide hormones [motilin, ghrelin, peptide YY (PYY), cholecystokinin (CCK), and glucagon-like peptide 1 (GLP-1)] in FD, which was correlated to intensities of the provoked symptoms. However, most studies are somewhat artificial as they used intraduodenal tube administration of selected nutrients, rather than ingestion of a true meal (27).

The aim of this review was to describe the current evidence on the role of gastrointestinal (GI) peptides in FGID, especially in FD and gastroparesis. We will also address implications for future applications or modulations of gastrointestinal peptides for FD and idiopathic and diabetic gastroparesis treatment.

## Methods

**Figure 1:**
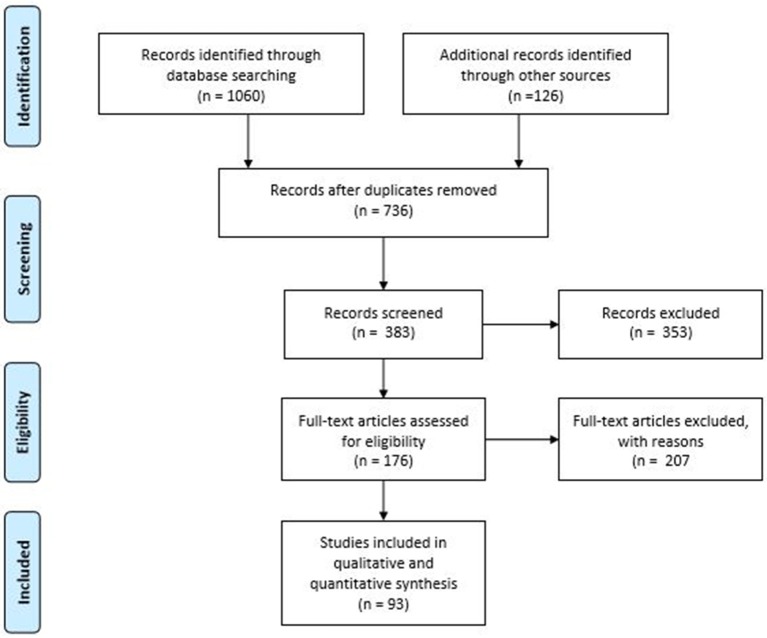
PRISMA flow chart of included studies in the systematic review.

We conducted a Pubmed and Medline search for papers, reviews, metanalyses, case series, and RCTs using the following keywords and their associations: functional dyspepsia, gastroparesis, gastrointestinal peptides, CCK, GLP-1, PYY, motilin, ghrelin, and dipeptidyl peptidase (Figure 1). We included also included preliminary evidence from abstracts belonging to main national and international gastroenterological meetings (e.g., United European Gastroenterology Week, Digestive Disease Week, Neurogastroenterology and Motility meetings, and the Belgian Gastroenterology week).

## Results

### Preliminary Consideration

**Figure 2:**
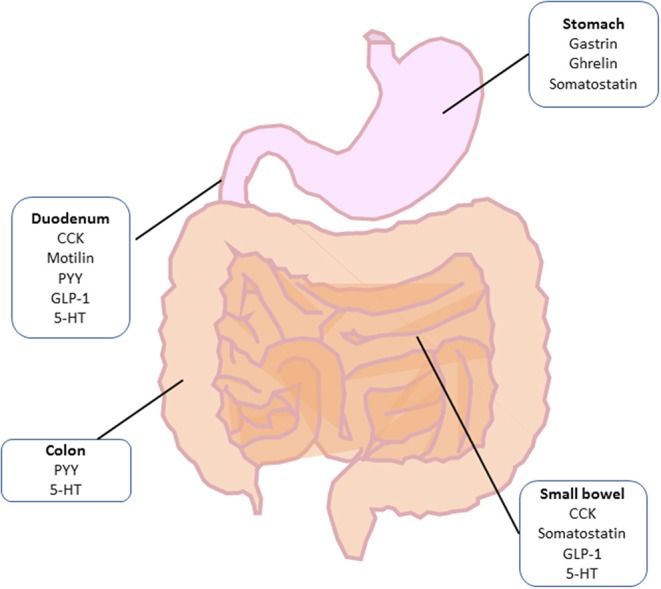
Overview of gastrointestinal peptides and their site of release.

Both in FD-PDS and in gastroparesis, symptoms are triggered by ingestion of a meal (28, 29). The release of gut peptides in response to nutrient intake is expected to be triggered sequentially, driven by the location of the entero-endocrine cells that are expressing them. Thus, nutrient arrival in the stomach is thought to affect the release of gastrin, ghrelin and potentially somatostatin, while duodenal exposure to nutrients may impact on the release of CCK, motilin, PYY, and GLP-1, among others (25–27). In addition, serotonin release is expected to occur when nutrients enter the duodenum (25) (Figure 2). The association between peptide levels and symptoms in FD and gastroparesis is summarized in Table 1.

**Table 1:** Summary findings on the link between gut peptides, functional dyspepsia, and gastroparesis.

**References**	**Peptide**	**Method**	**Subjects**	**Major findings**
Jonnson et al. (30)	Gastrin	Gastrin dosage	FD patients	No altered gastrin levels.
He et al. (31)	Gastrin	Gastrin dosage	FD patients with delayed gastric emptying and HV	Higher gastrin levels
Walecka-Kapica et al. (32)	Gastrin	Gastrin dosage	PDS and EPS patients and HV	Higher gastrin levels
Yoshikawa et al. (33)	Gastrin	Gastrin dosage	FD patients on H2-blockers	Gastrin levels do not predict H2-blockers response
He et al. (29)	Somatostatin	Plasma somatostatin dosage and mucosal expression	FD with normal/delayed gastric emptying and HV	No differences between FD and HV
Jonnson et al. (30)	Somatostatin	Plasma somatostatin dosage	FD with normal/delayed gastric emptying and HV	Higher somatostatin levels associated with higher symptoms' burden and higher heartburn severity scores; rapid, transient, somatostatin peak during a stress interview
Russo et al. (34)	Somatostatin	Plasma somatostatin dosage	42 PDS and 12 EPS patients	Somatostatin levels tendency to be lower in PDS vs. EPS, without reaching statistical significance
Katagiri et al. (35)	Somatostatin	Plasma somatostatin dosage	HV administered with Itopride	Acute increase of somatostatin levels
Foxx-Orenstein et al. (36)	Somatostatin	Somatostatin analog Octreotide administration	HV	Slowed gastric emptying, enhanced fasting gastric volumes and suppressed meal-induced volume increments
Yagi et al. (37)	Ghrelin	Ghrelin dosage	Gastroparesis and HV (multiple studies)	No significant difference between patients and HV
Kim et al. (38)	Ghrelin	Ghrelin dosage	PDS and EPS patients vs. HV	Significant correlation between ghrelin levels and symptom severity, namely epigastric pain in EPS, early satiation in PDS patients
Shindo et al. (39)	Ghrelin	Ghrelin dosage	PDS and EPS patients with NERD	Negative correlation between plasma ghrelin levels and gastric emptying rate in PDS but not with EPS patients
Takamori et al. (40)	Ghrelin	Ghrelin dosage	Dismotility-like dyspepsia patients (Rome II criteria) vs. HV	Lower Ghrelin levels vs. HV
Nishizawa et al. (41)	Ghrelin	Ghrelin dosage	FD patients vs. HV	Higher Ghrelin levels vs. HV
Pilichiewicz et al. (42)	Ghrelin	Ghrelin dosage	FD patients vs. HV with high-fat meal ingestion	Ingestion of the meal did not affect plasma ghrelin levels in FD vs. HV
Akamizu et al. (43)	Ghrelin	Ghrelin i.v. administration b.i.d. for 2 weeks	FD patients with loss of appetite	Significantly increased appetite and tendency to increased daily food intake in FD patients with loss of appetite
Arai et al. (44)	Ghrelin	Rikkunshito administration	FD patients	Improved upper gastrointestinal symptoms, correlating with increased plasm ghrelin levels
Suzuki et al. (45)	Ghrelin	Rikkunshito administration	*Helicobacter pylori*-infected participants with increased plasma ghrelin levels	Improved upper gastrointestinal symptoms
Gaddipati et al. (46)	Ghrelin	Ghrelin dosage	Idiopathic, diabetic and post-surgical Gastroparesis patients vs. HV gastroparesis	Increased ghrelin levels after sham feeding in HV and IG patients vs. diabetic and postsurgical gastroparesis
Tack et al. (47)	Ghrelin	Gastric emptying and meal-related symptoms evaluation	IG patients	Increased gastric emptying and improved symptoms
Murray et al. (48)	Ghrelin	Gastric emptying and meal-related symptoms evaluation	Diabetic gastroparesis patients	Increased gastric emptying
Binn et al. (49)	Ghrelin	Gastric emptying evaluation	Neurogenic Gastroparesis patients	Increased gastric emptying
Ejskjaer et al. (50)	Ghrelin	Ulimorelin (ghrelin agonist) i.v. administration	Diabetic gastroparesis patients	Increased gastric emptying
Heyland et al. (51)	Ghrelin	Ulimorelin (ghrelin agonist) i.v. administration vs. Metoclopramide	Critical ill patients with enteral feeding intolerance	Increased gastric emptying for both treatments, impossible differentiation
Ejskjaer et al. (52)	Ghrelin	TZP-102 (ghrelin agonist) Phase 2a study, 12 weeks study	Diabetic gastroparesis patients	Increased gastric emptying
Mc Callum et al. (53)	Ghrelin	TZP-102 ghrelin agonist) Phase 2b study, 12 weeks study	Diabetic gastroparesis patients	Failed to confirm Increased gastric emptying
Lembo et al. (54)	Ghrelin	Relamorelin injections	Diabetic gastroparesis patients	Reduced vomiting frequency/severity; accelerated gastric emptying
Camilleri et al. (55)	Ghrelin	Relamorelin injections	Diabetic gastroparesis patients	Accelerated gastric emptying
Russo et al. (34)	Motilin	Motilin dosage	PDS and EPS patients	Higher motilin levels in EPS vs. PDS patients
Labo et al. (56)	Motilin	Motilin dosage	FD patients with delayed gastric emptying	Absence of motilin levels fluctuations during the interdigestive state; gastric phase III contractions absence
Achem-Karam et al. (57)	Motilin	Motilin dosage	Diabetic gaastroparesis patients	Elevated and fluctuating motilin plasma levels during the interdigestive state; antral phase III activity is absent
Talley et al. (58)	Motilin	ABT-229 administration	FD patients with and without delayed gastric emptying	No significant symptoms improvement
Talley et al. (59)	Motilin	ABT-229 administration	Type 1 diabetes mellitus patients	No significant symptoms improvement
McCallum et al. (60)	Motilin	Mitemcinal	Patients with idiopathic and diabetic gastroparesis	Accelerates gastric empying
Mccallum et al. (61)	Motilin	Mitemcinal	Diabetic patients with gastroparesis symptoms	Symptoms relief vs. placebo
Cuomo et al. (62)	Motilin	Motilin dosage	HV	Contraction of proximal stomach, increases satiety
Deloose et al. (63)	Motilin	Camicinal	HV	Stimulates MMC and gastric emptying
Hellstrom et al. (64)	Motilin	Camicinal single dose administration (25, 50, or 125 mg)	Type 1 Diabetic patients with gastroparesis symptoms	significantly accelerated gastric emptying of solids by 125 mg dose
Barton et al. (65)	Motilin	Camicinal	Diabetic patients with gastroparesis symptoms	Significantly accelerated gastric emptying
Chapman et al. (66)	Motilin	Camicinal	Critical ill patients with enteral feeding intolerance	Camicinal single dose (50 mg) acceleratedgastric emptying and increased glucose absorption
Chiloiro et al. (67)	CCK	Standard solid-liquid meal (gastric emptying)	*H. pylori* associated dyspepsia patients	Significantly lower basal values compared to *H. pylori* negative patients
Bharucha et al. (27)	CCK	Intraduodenal dextrose and lipid adminstration	FD patients vs. HV	Correlation between plasma concentrations of CCK and provoked symptoms Early increase of CCK plasma levels
Barbera et al. (68)	CCK	Intraduodenal administration of lipids	FD patients	Increases sensitivity to gastric distention
Feinle et al. (69)	CCK	Duodenal lipid infusion + CCK-A antagonist dexloxiglumide	FD patients	Lipid increased plasma CCK levels Dexloxiglumide reduced gastric compliance Gastric distention relieved by dexloxiglumide
van Boxel et al. (70)	CCK	Duodenal perfusion	FD patients vs. HV	Mean mucosal CCK concentration was lower in FD patients
Feinle-Bisset et al. (71)	CCK	High (HF) and low (LF) fat yogurt	FD patients	Plasma CCK was higher after HF compared to LF
Rotondo et al. (72)	GLP-1	Dipeptidyl peptidase-4 inhibitor (vildagliptin)	HV	Inhibition of gastric accommodation and increased GLP-1 plasma levels
Mano et al. (73)	GLP-1	Hot water and broth (with rice)	HV	Rise in GLP-1 after ingestion of synthesized broth
Witte et al. (74)	GLP-1	Liquid meal	FD patients (EPS)	Similar GLP-1 levels to HV, correlation with nausea
Pilichiewicz et al. (42)	PYY	PYY dosage	FD patients vs. HV with high-fat meal ingestion	Lower postprandial PYY levels compared to HV
Witte et al. (74)	PYY	Liquid meal	FD patients (EPS)	PYY3-36 is correlated with the sensation of fullness
Tack et al. (75)	5-HT	Cisapride (5-HT4 agonist)	HV	Enhances gastric distension and accommodation
Kessing et al. (76)	5-HT	Prucalopride (5-HT4 agonist)	HV after a standardized meal	Accelerates gastric emptying in male volunteers
Carbone et al. (77)	5-HT	Prucalopride (5-HT4 agonist)	Patients with gastroparesis	Enhances gastric emptying
Netzer et al. (78)	5-HT	Ondansetron (5-HT3 antagonist)	HV	No effect on gastric emptying
Janssen et al. (79)	5-HT	Ondansetron (5-HT3 antagonist)	HV	No effect on gastric compliance, gastric tone
Van Oudenhove et al. (80)	5-HT	Busprione (5-HT1A agonist)	HV	Relaxation of the proximal stomach + decreases gastric emptying
Tack et al. (81)	5-HT	Busprione (5-HT1A agonist)	FD patients	Decreased symptoms + increased gastric accommodation
Geeraerts et al. (82)	5-HT	Acute tryptophan depletion	HV	Reduction in 5-HT levels in duodenum
Tack et al. (83)	5-HT	Paroxetine	HV	Enhances gastric accommodation
Janssen et al. (84)	5-HT	Citalopram (5-HT reuptake inhibitor)	HV	Preprandial gastric relaxation, lower postprandial volume increase + enhances liquid emptying
Jannsen et al. (85)	5-HT	Citalopram (5-HT reuptake inhibitor)	HV	Suppresses gastric phase 2 Stimulates intestinal phase 3
Wilmer et al. (86)	5-HT	Ondansetron (5-HT3 antagonist)	HV	Suppresses gastric component of phase 3
Chueng et al. (87)	5-HT	5-HT postprandial levels	FD patients	Decreased levels of 5-HT

### Gastrin

Gastrin is released by G-cells in the stomach and is a major stimulus for gastric acid secretion (25). As a group, FD patients do not seem to have altered gastrin levels according to a study of Jonsson et al. (30). However, in a study by He et al., FD patients with delayed gastric emptying had significantly higher gastrin levels (31). A recent study from Poland confirmed these findings, with elevated gastrin levels in both PDS and EPS (32). Use of acid suppressive therapy, often applied in FD as first-line therapy, may increase gastrin levels and it remains unclear to which extent the studies could rigorously exclude such confounder. In a relatively small study from Japan, gastrin serum level did not predict the response to H2 blocker therapy in FD (33).

### Somatostatin

Somatostatin is released in the stomach but also in the small bowel, and has a strong inhibitory effect on gastrointestinal motility and secretion (25). In the study by He et al., plasma somatostatin levels and mucosal expression of somatostatin in the antrum and the duodenum did not differ between health and FD, with normal or delayed emptying (31). The same was found in FD patients as a group in the study by Jonsson et al., but higher symptom burden was associated with higher fasting somatostatin levels in FD, and somatostatin levels were also correlated with heartburn severity scores (30). FD patients displayed a rapid, transient, somatostatin peak during a stress interview compared to matched controls (30). In a study by Russo et al., comparing gut peptide levels between 42 PDS and 12 EPS patients, somatostatin levels tended to be lower in PDS compared to EPS but this did not reach statistical significance (34). Itopride, a prokinetic agent with mixed dopamine-2 receptor and cholinesterase inhibitory actions, was reported to acutely increase somatostatin plasma levels (35). The somatostatin analog octreotide was reported to slow gastric emptying, enhance fasting gastric volumes and suppress meal-induced volume increments in healthy subjects (36). Clinical reports with somatostatin analogs in FD patients are lacking.

### Ghrelin

Ghrelin is produced by endocrine P/D1 cells in the stomach, with plasma levels that increase during fasting and decrease after food intake (25, 88). Ghrelin a 28 amino acid peptide which needs to have an octanoyl group attached to its third serine residue to be biologically active (25). Ghrelin levels are inversely related to body weight (89, 90) and decrease with increasing extent of gastric mucosal atrophy (91, 92). Several studies have investigated ghrelin release in FD and gastroparesis, and the findings are conflicting (38). Most studies found no difference in fasting ghrelin levels in FD compared to health (37, 39–41, 93, 94). However, in a small group of EPS and PDS patients compared to health, correlations were reported between ghrelin levels and symptom severity, in particular epigastric pain in EPS and early satiation in PDS (38). Shindo et al. found a negative correlation between plasma acylated ghrelin levels and gastric emptying rate in patients with PDS but not with EPS (39). Takamori et al. reported lower levels of des-acyl ghrelin (the inactive form after hydrolysis of the octanoyl group), in dysmotility-like dyspepsia according to the Rome II criteria (40) while Nishizawa et al. reported higher ghrelin levels in FD patients as a group (41). The ingestion of a high fat meal in FD patients did not differently affect the plasma ghrelin levels in FD compared to healthy subjects (42). Intravenous administration of ghrelin, twice daily for 2 weeks, significantly increased appetite and tended to increase daily food intake in FD patients with loss of appetite (43). Furthermore, Arai et al. observed a clear improvement in upper gastrointestinal symptoms in FD patients after administration of the Japanese Kampo medicine Rikkunshito, which increased plasma ghrelin levels (44). Suzuki et al. also showed Rikkunshito was effective among *H. pylori*-infected participants with increased plasma ghrelin levels (45).

Plasma ghrelin levels increased with sham feeding in healthy controls and patients with idiopathic gastroparesis but not in patients with diabetic or postsurgical gastroparesis, indicative of a role for intact vagal signaling in the control of ghrelin release (46). In pilot studies, acute intravenous administration of ghrelin enhanced gastric emptying rate in idiopathic and diabetic gastroparesis (47–49). In idiopathic gastroparesis patients, symptoms were also improved.

Subsequently, several ghrelin agonists have been studied, with a major focus on diabetic gastroparesis. The intravenously administered macrocylic peptidomimetic molecule ulimorelin, enhanced gastric emptying, and was subsequently mainly studied in critical care patients, with lack of differentiation from metoclopramide (50, 51). The orally administered TZP-102 showed promising results in phase 2a, but this was not confirmed in phase 2b (52, 53). Relamorelin, an injectable ghrelin receptor agonist, showed efficacy in diabetic gastroparesis patients with active vomiting symptoms in two placebo-controlled phase 2 studies and is being evaluated in phase 3 studies (54, 55).

### Motilin

Motilin is released from M-cells situated in the proximal duodenum during the fasted state, is a stimulus for strong antral contractions and has a hunger signaling function (25, 95). Several studies evaluated plasma motilin levels in FD and gastroparesis (34, 56, 96–98). FD patients as a group have comparable fasting plasma levels to those in health (95). Russo et al. reported higher fasting motilin plasma levels in EPS compared to PDS (34). In the same study, elevated CRF levels were also reported in PDS. The relevance of this finding is unclear. It is well-known that motilin plasma levels fluctuate with interdigestive motility and are maximal during gastric phase III (95). The study by Russo et al. did not correct for migrating motor complex (MMC) cycle, which could be a major confounder, as it is conceivable that PDS patients have less occurrence of gastric phase III (96).

In patients with FD and delayed gastric emptying, motilin plasma levels did not display the normal fluctuations during the interdigestive state and gastric phase III contractions were absent (56). In patients with diabetic gastroparesis, motilin plasma levels were elevated but still fluctuating during the interdigestive state, although antral phase III activity was absent (57, 98). In FD patients with unexplained loss of appetite, gastric phase III contractions are suppressed, suggesting low plasma levels, but these were not measured in this study (99).

Several macrolide antibiotics such as erythromycin and azithromycin have motilin receptor agonistic effects, and have a stimulatory effect on gastric emptying rate (100–102). The impact on symptoms, however, was often disappointing (101). A number of macrolides without antibiotics but with motilin receptor agonistic properties were developed for the treatment of FD and diabetic gastroparesis (103). However, invariably, they failed to provide significant symptomatic benefit in phase 2 studies and no agent progressed into phase 3 studies (58–61, 104). The main reasons that have been put forward to explain the lack of success in trials with motilin agonist drugs for gastroparesis have been the use of too high doses, which impact on gastric accommodation, and the use of long-acting agents which are prone to desensitization (62, 103).

Camicinal is a novel small molecule motilin receptor agonist with short half-life, which was shown to induce gastric phase III contractions during the fasting state and dose-dependently enhance gastric emptying rate (63, 64). In a phase 2 study, the lowest dose of camicinal significantly improved symptoms, confirming the therapeutic potential of this class of agents, whereas only the highest dose studied enhanced gastric emptying. Indicating that enhanced emptying rate does not underlie the symptom improvement (65). Camicinal was also studied in critical care patients, but the drug has not advanced to phase 3 in any indication (66).

### Cholecystokinin (CCK)

CCK is a brain-gut peptide released from I-cells in the upper small intestine upon food intake, especially after meals containing high fat or protein amounts (25). In *H. pylori* associated dyspepsia patients, significantly lower CCK basal values were demonstrated in comparison to *H. pylori* negative patients (67). Hyper responsiveness to CCK can be one of the pathophysiological pathways for the occurrence of symptoms in FD patients (105). A recent study showed a correlation between the release of gut peptide hormones as CCK and provoked symptoms after infusion of nutrients into the duodenum (27). However, in this study, intraduodenal tube administration of selected nutrients was used, rather than ingestion of a true meal. An early increase of CCK plasma levels was found, followed later by a rise of other peptides such as GLP-1 and PYY. Previously, it has also been shown that the intraduodenal infusion of fat may trigger symptoms as fullness and discomfort and to sensitize the stomach to gastric distension (68, 105). Duodenal lipids induce higher CCK levels in patients with FD compared to health, and the CCK-A receptor antagonist dexloxiglumide, was able to reduced sensitivity to gastric distension after lipid administration (69, 70, 106). However, ingestion of a low fat meal when patients perceived intake of a high fat meal (cognitive factors) did not significantly change the CCK level but was associated with higher symptom scores (71).

In addition, a CCK antagonist accelerated the gastric emptying rate which could lead to a benefit in both functional dyspepsia as gastroparesis patients (107). The improvement in gastric emptying probably involves an effect of CCK on capsaicin-sensitive vagal pathways (107). Infusion of CCK in healthy volunteers resulted in an increase in gastric compliance, but this was not confirmed in a study with FD patients (108). Unfortunately, in spite of a number of positive mechanistic observations, CCK-receptor antagonists were not further developed for the treatment of FD.

### Glucagon-Like Peptide 1 (GLP-1)

GLP-1, secreted by intestinal endocrine L-cells upon food intake, slows the gastric emptying in diabetes with a decrease in glycemia (108). In healthy controls, elevated GLP-1 plasma levels after administration of the Dipeptidyl peptidase-4 inhibitor vildagliptin, were associated with impaired gastric accommodation (72). In Japan, gastric emptying was measured in healthy subjects and increased significantly after ingestion of a broth with rice, which was accompanied by a significantly more rapid rise in plasma GLP-1 and glucose levels compared to rice with water (73). In an earlier study, it was shown that GLP-1 was correlated with nausea in a single meal experiment in FD patients subtype EPS as well as in healthy volunteers (74). This would be an interesting fact for the use of medication acting on the GLP-1 receptor for the treatment of gastroparesis patients with nausea as one of their main symptoms.

### Peptide YY (PYY)

PYY is a gut hormone secreted from endocrine L-cells in the gut mucosa, most prominently present in the ileum and the colon, and released into the circulation after ingestion of food (25, 109). As mentioned above, the intake of lipids is often a trigger for symptoms in FD. In FD patients, ingestion of a high fat meal was associated with lower postprandial PYY levels compared to healthy volunteers (42). In addition, PYY was found to be correlated with symptoms such as a sensation of fullness in EPS patients after a single drink test and a satiety test (74). However, based on the literature, little is known about the effect of PYY in FD patients.

### Serotonin (5-HT)

5-HT is also released by entero-endocrine cells in the gastrointestinal tract, in response to mechanical stimulation or the presence of nutrients or toxins (25, 110, 111). It has its effect via 14 known serotonin receptors, but we will focus on the most relevant ligands in this review. The role of 5-HT in upper gastrointestinal physiology remains unclear, due to a lack of suitable agonists and antagonists for human application (110). While 5-HT4 agonists enhance gastric accommodation and gastric emptying, 5-HT3 antagonists had no significant effect on these functions, and 5-HT_1A_ agonists enhance gastric accommodation and tend to slow gastric emptying (75–81). Alternative approaches to unravel a role for 5-HT in gastric sensorimotor function has been the use of tryptophan depletion (82) and the administration of selective serotonin reuptake inhibitors (SSRIs) (83, 84). Acute tryptophan depletion enhanced gastric accommodation, which was also observed with short-term SSRI use, while acute intravenous SSRI administration inhibited accommodation, suggesting that endogenous serotonin release serves to limit gastric accommodation (82–84). In terms of interdigestive gastric motility, acute intravenous SSRI administration suppresses gastric phase 3 while stimulating intestinal phase 3, and ondansetron also inhibited the occurrence of gastric phase 3 (85, 86).

Studies focusing on IBS have shown that circulating 5-HT levels rise after a meal, and that this rise is exaggerated in IBS with diarrhea and suppressed in IBS with constipation (112, 113). These studies used platelet-depleted plasma to measure circulating plasma levels of gastrointestinal origin, thereby eliminating the confounding effect of storage in thrombocytes. Similar studies in FD are lacking. One study measured plasma 5-HT in FD and found decreased basal and postprandial plasma compared to health (87). This is in agreement with a recent study reporting a decreased number of duodenal serotonin containing endocrine cells in FD patients (74).

Several 5-HT receptor agonists/antagonists, such as cisapride (5-HT4 agonist, 5-HT2, and 5-HT3 antagonist), tegaserod (5-HT4 and 5-HT1 agonist, 5-HT2a/b antagonist), mosapride (5-HT4 agonist, 5-HT3 antagonist) and revexepride (5-HT4 agonist) have been evaluated for the treatment of dyspepsia and gastroparesis, although not all studies show efficacy (114–116). A recent metanalysis showed that FD patients treated with serotonin receptor agonists have a significantly better symptom response compared to placebo (117) and the most recently published evidence indicates efficacy for prucalopride in idiopathic gastroparesis and emerging efficacy for velusetrag in gastroparesis (118, 119). Case series suggest potential benefit of the 5-HT_3_ antagonists granisetron or ondansetron for symptoms of nausea and vomiting in gastroparesis, but formal studies are lacking (120, 121).

## Summary and Conclusions

FD and gastroparesis, two of the most common FGIDs, are both characterized by upper GI symptoms. FD patients are subdivided in PDS and EPS patients, defined by symptoms as postprandial fullness, early satiety, epigastric pain, and epigastric burning. Patients with gastroparesis are characterized by nausea with or without vomiting, and often also similar symptoms as in FD, with a significantly delayed gastric emptying in the absence of mechanical obstruction. The most common subgroups are idiopathic and diabetic gastroparesis. The pathophysiology of both FGIDs is based on a combination of motility disturbances, visceral hypersensitivity, low grade mucosal immune activation, and altered processing of gut-brain-signals. Recent observations support a new pathophysiological model in at least subsets of patients with FD and gastroparesis, which involves visceral hypersensitivity to nutrients. Nutrient sensing occurs in the stomach and duodenum and is signaled to the brain through neural pathways, but especially through the release of gut peptides, which was shown in some studies to be correlated with symptoms in FD and gastroparesis.

In this review, the effect of peptides as gastrin, somatostatin, and ghrelin, all released by endocrine cells in the stomach, and of motilin, CCK, GLP-1, PYY and 5-HT, secreted in the duodenum, was summarized. Previous studies showed contradictory results regarding an increase in peptide levels in FD patients compared to health, but the impact of confounders, as the use of acid suppressive therapy for gastrin, the impact of MMC cycle for motilin, and the accumulation of 5-HT in thrombocytes, was not taken into account (28–30, 50). In most studies, the levels of somatostatin, ghrelin, and motilin did not differ between healthy volunteers and FD patients, however higher symptom burden was often correlated with higher peptide levels (28, 29, 36, 48). Nevertheless, most of these studies are limited by small sample sizes. Furthermore, a study by Russo et al. showed a trend toward higher somatostatin and motilin levels in EPS patients compared to PDS patients (32). However, the effect of gut peptides was mainly analyzed in FD patients as a group compared to healthy controls and only rarely in terms of EPS vs. PDS subgroups. In addition, little is known about the relation of gut peptides in FD patients fulfilling Rome IV criteria. *H. pylori* associated dyspepsia patients were shown to have lower CCK levels compared to *H.pylori* negative patients (67).

In patients with FD and gastroparesis, the correlation of gut peptides and gastric emptying was studied. Previously, a negative correlation was found between acylated ghrelin and gastric emptying (39). Intravenous administration of ghrelin increased the appetite in FD and enhanced gastric emptying and symptoms in idiopathic gastroparesis (43, 47–49). In addition, intraduodenal administration lipid administration provoked FD symptoms whose severity was correlated with CCK levels (27). Nevertheless, studies in which gut peptides are examined after eating a standard meal with an analysis on symptoms and motility disturbances, are lacking.

Based on the literature, low grade inflammation with increased mast cell and eosinophil count would underlie in the pathophysiological mechanisms of FGIDs and lead to an impaired barrier function. Duodenal factors, such as nutrients, may play a role in the activation of those eosinophils and mast cells. Therefore, it would be interesting to further investigate the effect of nutrients or diets on the release of GI peptides and evaluate this as a potential treatment option for FD or gastroparesis. Drugs acting on peptide receptors have already been tested in both groups, but is the scope of the available data is limited. Ghrelin agonists such as ulimorelin, relamorelin, and TZP-102, as well as 5-HT4 agonists and CCK antagonists all showed promising results in terms of improvement of the gastric emptying (50, 52–55, 66, 117–119). In addition, the use of motilin receptor agonists (macrolide antibiotics and camicinal) enhanced the gastric emptying, but there the link with improvement of symptoms is less predictable (66, 101). Serotonin agonists have a potential to improve symptoms in both FD and idiopathic gastroparesis (114, 117–119). Drugs acting on the GLP-1 and on the PYY receptors deserve further investigation, because of the link between GLP-1 release and nausea, and the link between PYY release and postprandial fullness (74).

In summary, there is a clear need for in-depth evaluation of release of GI peptides after a standard meal in larger sample sizes of Rome IV PDS and EPS and gastroparesis patients. This should be complemented with detailed studies of drugs altering the level of GI peptides or their effect on their receptors.

## Data Availability Statement

The raw data supporting the conclusions of this article will be made available by the authors, without undue reservation, to any qualified researcher.

## Author Contributions

KV, ES, and JT drafted the manuscript. All authors made edits and corrections and reviewed and approved the final version of the test.

### Conflict of Interest

JT has given Scientific advice to AlfaWassermann, Allergan, Christian Hansen, Danone, Grünenthal, Ironwood, Janssen, Kiowa Kirin, Menarini, Mylan, Neutec, Novartis, Noventure, Nutricia, Shionogi, Shire, Takeda, Theravance, Tramedico, Truvion, Tsumura, Zealand, and Zeria pharmaceuticals and has served on the Speaker bureau for Abbott, Allergan, AstraZeneca, Janssen, Kyowa Kirin, Menarini, Mylan, Novartis, Shire, Takeda, Truvion, and Zeria. The remaining authors declare that the research was conducted in the absence of any commercial or financial relationships that could be construed as a potential conflict of interest.
